# Collateral damage: Cardiovascular and respiratory implications of tear gas deployment during peaceful protest

**DOI:** 10.1016/j.toxrep.2025.102166

**Published:** 2025-11-17

**Authors:** Konstantine Chakhunashvili, Gela Gunashvili, Nino Jobava, George Chakhunashvili, Davit G. Chakhunashvili

**Affiliations:** a Caucasus University, Tbilisi, Georgia; b Pediatric Cardio-Rheumatologist, Georgian Pediatric Cardiology Association, Tbilisi, Georgia; c Alte University, International School of Medicine, Tbilisi, Georgia

**Keywords:** Tear gas, ECG, Capillaroscopy

## Abstract

**Background:**

In December 2024, large-scale protests in front of Georgia’s Parliament were met with crowd-control measures involving the widespread use of tear gas and pepper spray, mixed with water. This study examined whether protest participants exposed to these agents exhibited electrocardiographic, capillaroscopic, or hematologic abnormalities, and explored associations with allergy status, mask use, and attendance frequency.

**Methods:**

An observational case-control study was conducted from January 9 to March 1, 2025. Of 347 protest participants surveyed, 69 underwent clinical evaluation. A control group of 31 unexposed individuals was recruited. Participants received ECGs, capillaroscopy, complete blood count (CBC), and coagulogram testing. Data were analyzed using chi-square tests, eta coefficients, and t-tests (p < 0.05).

**Results:**

ECG abnormalities—including P-wave (p < 0.001), QRS complex (p = 0.035), and T-wave (p = 0.012) changes—were significantly more frequent in the exposed group. Right bundle branch block and T-wave inversions were particularly notable. Capillaroscopy showed more non-specific and sclerodermal abnormalities in the exposed group, though not statistically significant. Allergy status was linked to higher symptom burden, while mask use and attendance frequency were not predictive. Laboratory parameters were largely normal. Two respiratory cases—hypersensitivity pneumonitis and unresolved pneumonia—were clinically linked to exposure.

**Conclusion:**

Exposure to CS gas was associated with significant ECG changes, indicating potential cardiopulmonary effects. Clinical patterns and rare respiratory cases warrant re-evaluation of chemical agent use, improved oversight, and long-term studies to assess chronic health risks in exposed populations.

## Highlights

• Tear gas exposure is associated RBBB and T-wave inversions on ECG.
• Two incidences of hypersensitivity pneumonitis.
• Mask type or use showed no link to symptom incidence.
• Capillaroscopy changes more frequent but not statistically significant.
• Findings demand stricter oversight and long-term study of CS gas effects.

## Graphical Abstract

**Figure:**
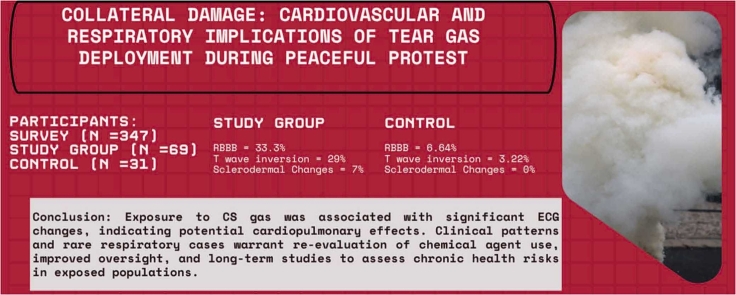

## 1 Introduction

In 2024, Georgia experienced a period of heightened political tension, largely triggered by the government’s decision to reintroduce the controversial “Russian law,” a legislative initiative that had already provoked widespread unrest just a year earlier. Following renewed public protests, the government responded with increasing authoritarian measures, including the use of police force, the mobilization of criminal groups who physically assaulted citizens near their homes, and an orchestrated campaign of intimidation involving threatening phone calls to tens of thousands of individuals. These developments culminated in a deeply contentious general election, widely criticized for failing to meet democratic standards. Allegations included multiple voting, voter intimidation, ballot manipulation, and an insufficient number of polling stations for over 150,000 Georgian emigrants residing in Western countries. This, in turn, reignited a new wave of nationwide protests. The situation escalated dramatically following a speech at Georgian Dream (GD) party office, in which the suspension of Georgia’s EU accession process was announced in retaliation to a European Parliament resolution calling for sanctions against the inner circle of Bidzina Ivanishvili—a Russian-linked oligarch—and members of the ruling GD party. On November 28, mass protests erupted across the country. In response, riot police were deployed and employed aggressive crowd control tactics, including water cannons mixed with pepper spray, handheld pepper sprays, and large quantities of tear gas, which they had used up until 7th on December 2024 [1], [2], [3], [4], [5], [6].

Many individuals reported experiencing serious signs and symptoms during the protests; however, authorities did not disclose the specific chemical agents used. Through field observation, there were multiple reports of GL-202 tear gas canisters at the demonstration sites. Further investigation revealed that these canisters are manufactured by Condor, a Brazilian arms producer, and contain ortho-chlorobenzylidene malononitrile (CS gas)—a potent lachrymatory agent—at a notably high payload of 23 g per capsule. According to the manufacturer’s specifications, the GL-202 is intended for indirect use and should not be aimed directly at individuals [7] (supplemental file 1–3). Nevertheless, at least one confirmed case involved a demonstrator who sustained severe facial trauma after being struck by a gas canister during the dispersal operation [8].

Given the scale and intensity of chemical agent use during the 2024 protests—particularly the deployment of GL-202 tear gas canisters containing high doses of CS—concerns emerged regarding the potential short- and long-term health consequences for exposed individuals. CS gas, while typically classified as a “non-lethal” riot control agent, has well-documented irritant effects on the respiratory, cardiovascular, and integumentary systems. However, limited research exists on its systemic impact under real-world conditions, especially in dense urban environments with repeated exposure and potential for vulnerable populations to be affected. In this context, we hypothesized that individuals exposed to tear gas during the 2024 demonstrations in Tbilisi would exhibit a significantly higher prevalence of electrocardiographic, CBC and coagulogram abnormalities—compared to non-exposed individuals. Additionally, we explored whether allergy status, frequency of attendance, and facemask use influenced symptom severity or physiological findings, and whether capillaroscopic abnormalities might indicate subclinical microvascular involvement.

## 2 Materials and methods

### 2.1 Study design and data collection

The study was designed as an observational case-control study and conducted between January 9 and March 1. We developed a structured questionnaire to survey individuals who participated in the demonstrations that began in Georgia after November 28, 2024. Respondents provided anthropometric data, allergy and smoking status, symptoms or signs developed during the chemical weapon demloyment (presentation and persistance beyond 30 days), type (and use) of facemask (none, non-industrial semi-face mask, industrial semi-face mask, industrial full-face mask), and the number of demonstrations participants attended during which riot police deployed chemical agents. In total, 347 unique survey responses were obtained; all respondents were then invited to undergo a medical examination, which included measurement of body weight, height, blood pressure, oxygen saturation, electrocardiography (ECG), capillaroscopy, complete blood count (CBC), and coagulogram. A total of 69 participants consented to and completed the examination, forming the study group. Criteria for inclusion were: a) > 18 years, b) were not a subject of physical abuse from the police, and c) attended demonstrations between November 28th and December 7th, including those during which the chemical agents were not deployed.

To enable comparison of capillaroscopic and ECG findings, we also enrolled a control group of 31 individuals who were (a) > 18 years old, (b) lived outside the Rustaveli Avenue district and (c) had not participated in any demonstrations.

Non-numerical ECG findings were recorded under the following categories: rhythm, axis, P wave, QRS complex, ST segment, and T wave; for each, the presence or absence of abnormalities was noted.

Capillaroscopy is a non-invasive diagnostic technique used to visualize the microcirculation, particularly the capillaries at the nailfold. It is commonly employed to assess capillary morphology, density, and flow patterns. Capillaroscopy provides valuable insights into vascular health and systemic disease activity. Capillaroscopic observations were similarly categorized as: transparency, capillary flow, overall architecture, avascular zones, capillary swelling, semi-quantitative rating scale, and an integrated summary conclusion. To calculate capillaroscopic parameters, which facilitated the identification of specific patterns and the determination of a semi-quantitative score, we used the Spanish software Capillary.io (https://app.capillary.io/).

Complete blood count (CBC) parameters and coagulogram measures—including fibrinogen, thrombin time (TT), activated partial thromboplastin time (aPTT), prothrombin time (PT), and international normalized ratio (INR)—were first recorded as continuous variables. Each result was subsequently classified as “normal” or “abnormal” according to established laboratory reference ranges.

Symptoms and signs were recorded dichotomously (yes/no). An additional cumulative score was calculated by assigning “yes” = 1 and “no” = 0, then summing across all items (a score of 0 indicating no reported symptoms or signs). Participants were asked to report both the symptoms and signs they were experiencing at the time of the survey and those they had experienced at the onset of chemical compound exposure.

### 2.2 Sample size and study group strength

Given the ethical and logistical constraints of recruiting protest-exposed individuals in a politically sensitive setting, we employed a convenience sampling strategy. A total of 347 respondents completed the structured questionnaire, of whom 69 consented to and completed the full clinical evaluation, forming the study group. While not calculated prospectively due to the urgent and observational nature of the research, post hoc effect size calculations suggest that a sample of ≥ 60 exposed individuals provides sufficient sensitivity to detect moderate-to-large effects (Cohen’s d > 0.6 or risk difference > 20 %) with 80 % power at an alpha level of 0.05. The control group (n = 31), though smaller, was matched for key demographic factors and served as a reasonable comparison group under real-world constraints. Future studies with larger cohorts will be necessary to validate subgroup effects and assess long-term outcomes.

### 2.3 Statistical analysis

Statistical analyses were performed in SPSS version 26. Continuous variables were examined using Pearson’s correlation coefficient and linear regression. Associations between continuous and categorical variables were assessed with the eta coefficient, while relationships among categorical variables were tested via chi-square analysis. Group differences in means were evaluated using independent-samples *t*-tests or one-way Analysis of Variance, as appropriate. All tests were two-tailed, and statistical significance was defined as p < 0.05 with a 95 % confidence interval.

## 3 Results

### 3.1 General data

Among 347 respondents (aged 18–70 years), 237 (68.3 %) were female and 110 (31.7 %) were male. A total of 193 (55.6 %) reported current smoking, while 154 (44.4 %) were non-smokers. Of 69 people who underwent testing we 27 were male (39.1 %) and 42 were female (60.9 %), 29 were non smokers (42 %) and 40 were smokers (58 %). All 69 participants were exposed to the tear gas. Regarding allergy status, 110 (31.7 %) indicated they had allergies and 237 (68.3 %) reported none. During the violent dispersion of demonstrations, the most frequent developed symptom was headache (68.3 %), followed by cough (63.7 %). Thirty days or more after exposure, fatigue was reported most often persisting symptom (44.1 %), followed by headache (40.9 %) and cough (33.7 %), whilst psychological sequelae exhibited minimal reduction over time, decreasing from 45.5 % to 36 % (Table 1). In the control group (n = 31), 16 participants (51.6 %) were smokers and 15 (48.4 %) were non-smokers; 14 (45.2 %) were female and 17 (54.8 %) were male.

**Table 1:** The table presents prevalence of signs and symptoms that manifested both during exposure to the chemical compounds and at least 30 days after their use.

			Frequency	Percent
Cough - During violent dispersion of demonstrations		No	126	36.3
		Yes	221	63.7
		Total	347	100.0
			Frequency	Percent
Shortness of breath - During violent dispersion of demonstrations		No	182	52.4
		Yes	165	47.6
		Total	347	100.0
			Frequency	Percent
Rhinorrhea - During violent dispersion of demonstrations		No	211	60.8
		Yes	136	39.2
		Total	347	100.0
			Frequency	Percent
Epistaxis - During violent dispersion of demonstrations		No	292	84.1
		Yes	55	15.9
		Total	347	100.0
			Frequency	Percent
Nausea - During violent dispersion of demonstrations		No	247	71.2
		Yes	100	28.8
		Total	347	100.0
			Frequency	Percent
Vomiting - During violent dispersion of demonstrations		No	312	89.9
		Yes	35	10.1
		Total	347	100.0
			Frequency	Percent
Abdominal pain - During violent dispersion of demonstrations		No	269	77.5
		Yes	78	22.5
		Total	347	100.0
			Frequency	Percent
Headache - During violent dispersion of demonstrations		No	110	31.7
		Yes	237	68.3
		Total	347	100.0
			Frequency	Percent
High blood pressure - During violent dispersion of demonstrations		No	283	81.6
		Yes	64	18.4
		Total	347	100.0
			Frequency	Percent
Heart palpitation - During violent dispersion of demonstrations		No	157	45.2
		Yes	190	54.8
		Total	347	100.0
			Frequency	Percent
Fatigue - During violent dispersion of demonstrations		No	133	38.3
		Yes	214	61.7
		Total	347	100.0
			Frequency	Percent
Fatigue - During violent dispersion of demonstrations		No	133	38.3
		Yes	214	61.7
		Total	347	100.0
			Frequency	Percent
Muscle and/or joint pain - During violent dispersion of demonstrations		No	200	57.6
		Yes	147	42.4
		Total	347	100.0
			Frequency	Percent
Eye and/or vission sequale - During violent dispersion of demonstrations		No	166	47.8
		Yes	181	52.2
		Total	347	100.0
			Frequency	Percent
Skin Disorders (rash, - During violent dispersion of demonstrations		No	199	57.3
		Yes	148	42.7
		Total	347	100.0
			Frequency	Percent
Psychological sequalae (Anxiety, Panic attacks, PTSD etc.) - During violent dispersion of demonstrations		No	189	54.5
		Yes	158	45.5
		Total	347	100.0
		Frequency	Percent	
Cough - 30 + days after violent dispersion of the demonstration	No	230	66.3	
	Yes	117	33.7	
	Total	347	100.0	
		Frequency	Percent	
Shortness of breath - 30 + days after violent dispersion of the demonstration	No	247	71.2	
	Yes	100	28.8	
	Total	347	100.0	
		Frequency	Percent	
Rhinorrhea - 30 + days after violent dispersion of the demonstration	No	271	78.1	
	Yes	76	21.9	
	Total	347	100.0	
		Frequency	Percent	
Epistaxis - 30 + days after violent dispersion of the demonstration	No	327	94.2	
	Yes	20	5.8	
		
	Total	347	100.0	
		Frequency	Percent	
Nausea - 30 + days after violent dispersion of the demonstration	No	320	92.2	
	Yes	27	7.8	
	Total	347	100.0	
		Frequency	Percent	
Vomiting - 30 + days after violent dispersion of the demonstration	No	339	97.7	
	Yes	8	2.3	
	Total	347	100.0	
		Frequency	Percent	
Abdominal pain - 30 + days after violent dispersion of the demonstration	No	319	91.9	
	Yes	28	8.1	
	Total	347	100.0	
		Frequency	Percent	
Diarrhea - 30 + days after violent dispersion of the demonstration	No	327	94.2	
	Yes	20	5.8	
	Total	347	100.0	
		Frequency	Percent	
Headache - 30 + days after violent dispersion of the demonstration	No	205	59.1	
	Yes	142	40.9	
	Total	347	100.0	
		Frequency	Percent	
High blood pressure - 30 + days after violent dispersion of the demonstration	No	311	89.6	
	Yes	36	10.4	
	Total	347	100.0	
		Frequency	Percent	
Heart palpitation - 30 + days after violent dispersion of the demonstration	No	241	69.5	
	Yes	106	30.5	
	Total	347	100.0	
		Frequency	Percent	
Fatigue - 30 + days after violent dispersion of the demonstration	No	194	55.9	
	Yes	153	44.1	
	Total	347	100.0	
		Frequency	Percent	
Muscle and/or joint pain - 30 + days after violent dispersion of the demonstration	No	261	75.2	
	Yes	86	24.8	
	Total	347	100.0	
		Frequency	Percent	
Eye and vission sequale - 30 + days after violent dispersion of the demonstration	No	248	71.5	
	Yes	99	28.5	
	Total	347	100.0	
		Frequency	Percent	
Skin Disorders (rash, itching and etc.) - 30 + days after violent dispersion of the demonstration	No	290	83.6	
	Yes	57	16.4	
	Total	347	100.0	
		Frequency	Percent	
Psychological sequalae (Anxiety, Panic attacks, PTSD etc.) - 30 + days after violent dispersion of the demonstration	No	222	64.0	
	Yes	125	36.0	
	Total	347	100.0	

### 3.2 ECG results

Rhythm: In the control group, 30 patients (96.78 %) had sinus rhythm, and 1 patient (3.22 %) had first-degree atrioventricular (AV) block. In the study group, 67 patients (97.11 %) had sinus rhythm, and 2 patients (2.89 %) presented with premature ventricular contractions. No other rhythm abnormalities were identified. There was no statistically significant difference between the groups, χ²(1) = 0.007, p = .933, 0.31 % (95 % CI [−7.23 %, 13.43 %]).

P wave abnormalities: In the control group, 3 patients (9.67 %) had variable P waves, while 28 (90.33 %) were normal. In the study group, 23 patients (33.3 %) had variable P waves, 2 (2.9 %) had peaked P waves, 10 (14.5 %) had inverted P waves, and 34 (49.3 %) had normal P waves. The overall proportion of abnormalities was significantly higher in the study group, χ²(1) = 15.77, p < .001, 42.03 % (95 % CI [22.91 %, 55.06 %]).

QRS complex abnormalities: In the control group, right bundle branch block (RBBB) was observed in 2 cases (6.64 %), 1 case had a pathological Q wave (3.22 %), and 2 cases had poor R wave progression (6.64 %), while 26 (83.5 %) were normal. In the study group, 23 patients (33.3 %) had RBBB, 1 (1.4 %) had a pathological Q wave, 2 (2.9 %) had deformed Q waves, and 43 (62.3 %) had normal findings. The overall difference in QRS abnormalities was statistically significant, χ²(1) = 4.439, p = .035, of 21.2 % (95 % CI [1.59 %, 36.14 %]). Specifically, the prevalence of RBBB was also significantly higher in the study group, χ²(1) = 8.020, p = .005, 26.66 % (95 % CI [9.20 %, 39.33 %]).

ST segment changes: In the control group, ST segment depression was observed in 1 patient (3.22 %) and early repolarization in 4 patients (12.90 %), while 26 (83.88 %) had normal ST segments. In the study group, 2 patients (2.89 %) had ST segment elevation, and 67 (97.11 %) were normal. Although more abnormalities were detected in the control group, the comparison of ST elevation alone did not reveal a statistically significant difference, χ²(1) = 0.905, p = .341, with a difference of 2.89 % (95 % CI [−8.33 %, 9.95 %]).

T wave abnormalities: In the control group, T wave inversion, peaked T wave, and biphasic T wave were each observed in 1 patient (3.22 %), and flattened T waves in 4 patients (12.90 %). In the study group, T wave inversion was observed in 20 patients (29 %), peaked T wave in 1 (1.4 %), and flattened T waves in 2 (2.9 %), with 46 patients (66.7 %) showing normal T waves. The difference in T wave inversion was statistically significant, χ²(1) = 6.308, p = .012, with a difference of 19 % (95 % CI [4.94 %, 29.94 %]). No significant differences were found for flattened T waves, χ²(1) = 3.755, p = .053, 10.0 % (95 % CI [−0.50 %, 26.09 %]), or for peaked T waves, χ²(1) = 0.364, p = .546, 1.82 % (95 % CI [−5.00 %, 14.84 %]).

In the study group, no association was observed between smoking and P-wave abnormalities (χ²(3) = 1.85, p = .604), QRS abnormalities (χ²(3) = 1.56, p = .668), or T-wave abnormalities (χ²(3) = 0.85, p = .012); the unusually low p-value for T-wave relative to the small χ² statistic should be verified. Similarly, facemask type was not associated with P-wave (χ²(3) = 8.74, p = .461), QRS (χ²(3) = 7.59, p = .576), or T-wave (χ²(3) = 10.71, p = .296) abnormalities. No association was found between days spent at demonstrations involving chemical agents and P-wave abnormalities (η(15) = 0.27, p = .534), QRS abnormalities (η(15) = 0.25, p = .570), or T-wave abnormalities (η(15) = 0.30, p = .736). Likewise, BMI showed no association with P-wave abnormalities (η(15) = 0.96, p = .820), QRS abnormalities (η(15) = 0.96, p = .517), or T-wave abnormalities (η(15) = 0.96, p = .501). Ensure that the reported p-values and effect-size estimates are consistent with the original statistical outputs.

### 3.3 Capillaroscopy results

**Fig. 1:**
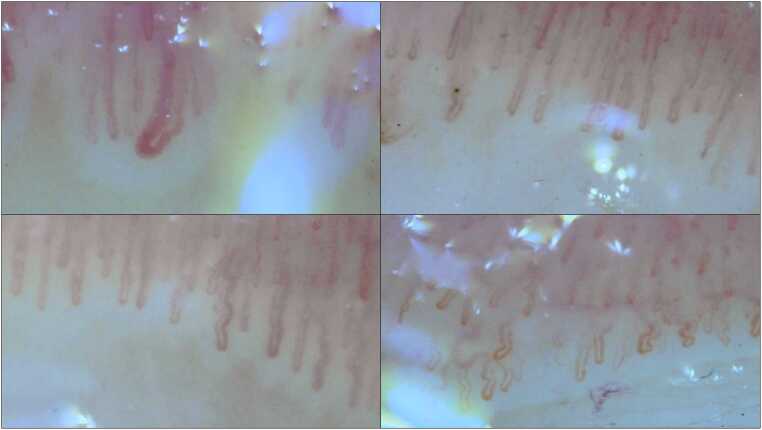
Microscopic images showing capillaroscopic changes with sclerodermal patterns in four patients exposed to chemical agents. Capillaroscopic changes consistent with a sclerodermal pattern were observed in four patients; representative images from all four cases are shown in this figure. (Magnification = X200).

Of 31 control participants, 3 (9.7 %) were excluded due to inability to visualize the capillaries; among the remaining 28, 16 (57.1 %) exhibited a normal capillaroscopic pattern and 12 (42.9 %) exhibited non-specific abnormalities. In the study group, 12 of 69 participants (17.4 %) were excluded for the same reason, leaving 57: 22 (38.6 %) demonstrated a normal pattern, 31 (54.4 %) showed non-specific changes, and 4 (7.0 %) showed sclerodermal changes (Fig. 1). No statistically significant difference was detected in the proportion of non-specific changes between groups (11.55 % (95 % CI [−10.59 %, 31.97 %], χ²(1) = 0.99, p = .320)), and in sclerodermal changes (7.0 % (95 % CI [−5.79 %, 16.68 %], χ²(1) = 2.03, p = .154)).

In the control group, 5 participants (17.9 %) exhibited < 33 % capillary abnormalities, 20 (71.4 %) exhibited 33–66 % abnormalities, and 3 (10.7 %) exhibited > 66 % abnormalities. In the study group, 15 (26.3 %) had < 33 %, 32 (56.1 %) had 33–66 %, and 10 (17.6 %) had > 66 % abnormalities. There were no significant differences between groups for the 33–66 % category (difference 6.49 % [95 % CI −11.80 % to 20.33 %], χ²(1) = 0.60, p = .439) or for the > 66 % category (difference 14.93 % [95 % CI −7.17 % to 33.52 %], χ²(1) = 1.74, p = .187).

### 3.4 CBC, coagulogram and blood pressure

During data analysis, almost all of participants in the study group exhibited laboratory values within standard reference ranges. The few abnormalities observed were minor; therefore, we did not perform CBC or coagulogram testing in the control group and did not conduct statistical comparisons of blood pressure between groups.

### 3.5 Survey analysis

**Fig. 2:**
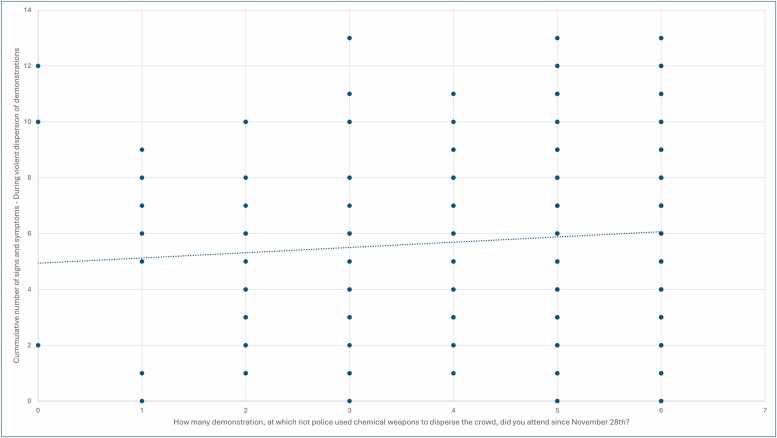
The figure illustrates a linear relationship between the number of days attended and the cumulative number of signs and symptoms reported in close proximity to the time of chemical dispersion.

Days of demonstrations attended: The only association observed was a weak positive correlation between the cumulative number of signs (Fig. 2) and symptoms and the frequency of abdominal pain experienced in close proximity to violent dispersal of demonstrations (Table 2).

**Table 2:** depicts correlation analysis results between various variables that were collected through the survey.

	Days of demonstrations attended during which riot police deployed chemical agents	BMI	Smoking status	Use of masks	Allergy status
Cummulative number of signs and symptoms - During violent dispersion of demonstrations	R(345) = .09 R² = .01 F = 2.88 p = .045	R(345) = .06 R² = .00 F = 1.27 p = .261	η (13) = 0.17, p = .147	η (13) = 0.17, p = .147	η (13) = 0.21, p = .007
Cummulative number of signs and symptoms - 30 + days after violent dispersion of the demonstration	R(345) = .03 R² = .00 F = .31 p = .288	R(345) = .03 R² = .00 F = 0.46 p = .249	η (13) = 0.00, p = .675	η (36) = 0.22, p = .431	η (13) = 0.21, p = .072
Cough - During violent dispersion of demonstrations	η (345) = 0.05, p = .274	η (345) = 0.06, p = .483	χ²(1) = 10.01, p = .002	χ²(3) = 2.56, p = .464	χ²(1) = 1.41, p = .236
Shortness of breath - During violent dispersion of demonstrations	η (345) = 0.20, p = .275	η (345) = 0.94, p = .300	χ²(1) = 3.98, p = .046	χ²(3) = 2.11, p = .550	χ²(1) = 1.176, p = .278
Rhinorrhea - During violent dispersion of demonstrations	η (345) = 0.09, p = .971	η (345) = 0.04, p = .501	χ²(1) = 1.40, p = .236	χ²(3) = 0.45, p = .929	χ²(1) = 1.33, p = .248
Epistaxis - During violent dispersion of demonstrations	η (345) = 0.11, p = .230	η (345) = 0.04, p = .448	χ²(1) = 2.56, p = .110	χ²(3) = 0.88, p = .830	χ²(1) = 0.59, p = .442
Nausea - During violent dispersion of demonstrations	η (345) = 0.04, p = .136	η (345) = 0.13, p = .015	χ²(1) = 0.32, p = .570	χ²(3) = 7.19, p = .066	χ²(1) = 0.01, p = .939
Vomiting - During violent dispersion of demonstrations	η (345) = 0.10, p = .474	η (345) = 0.06, p = .253	χ²(1) = 3.94, p = .047	χ²(3) = 0.715, p = .870	χ²(1) = 2.24, p = .135
Abdominal pain - During violent dispersion of demonstrations	η (345) = 0.15, p = .029	η (345) = 0.03, p = .500	χ²(1) = 2.93, p = .087	χ²(3) = 3.77, p = .287	χ²(1) = 0.124, p = .725
Diarrhea - During violent dispersion of demonstrations	η (345) = 0.02, p = .699	η (345) = 0.02, p = .669	χ²(1) = 3.16, p = .075	χ²(3) = 2.93, p = .087	χ²(1) = 3.74, p = .053
Headache - During violent dispersion of demonstrations	η (345) = 0.09, p = .998	η (345) = 0.03, p = .553	χ²(1) = 0.08, p = .784	χ²(3) = 1.34, p = .718	χ²(1) = 3.81, p = .051
High blood pressure - During violent dispersion of demonstrations	η (345) = 0.08, p = .162	η (345) = 0.18, p = .001	χ²(1) = 3.18, p = .074	χ²(3) = 7.84, p = .049	χ²(1) = 1.22, p = .270
Heart palpitation - During violent dispersion of demonstrations	η (345) = 0.08, p = .162	η (345) = 0.045,p = .408	χ²(1) = 1.88, p = .170	χ²(3) = 2.22, p = .527	χ²(1) = 1.22, p = .269
Fatigue - During violent dispersion of demonstrations	η (345) = 0.05, p = .354	η (345) = 0.05, p = .402	χ²(1) = 4.91, p = .027	χ²(3) = 0.66, p = .219	χ²(1) = 0.263, p = .608
Muscle and/or joint pain - During violent dispersion of demonstrations	η (345) = 0.07, p = .226	η (345) = 0.07, p = .206	χ²(1) = 2.51, p = .113	χ²(3) = 3.55, p = .314	χ²(1) = 0.00, p = .925
Eye and/or vission sequale - During violent dispersion of demonstrations	η (345) = 0.17, p = .947	η (345) = 0.01, p = .871	χ²(1) = 0.00, p = .943	χ²(3) = 3.72, p = .293	χ²(1) = 11.40, p = .001
Skin Disorders (rash, and etc.) - During violent dispersion of demonstrations	η (345) = 0.14, p = .249	η (345) = 0.00, p = .936	χ²(1) = 0.00, p = .881	χ²(3) = 0.19, p = .729	χ²(1) = 7.95, p = .005
Psychological sequalae (Anxiety, Panic attacks, PTSD etc.) - During violent dispersion of demonstrations	η (345) = 0.05, p = .335	η (345) = 0.09, p = .084	χ²(1) = 4.82, p = .028	χ²(3) = 1.44, p = .696	χ²(1) = 1.18, p = .171
Cough - 30 + days after violent dispersion of the demonstration	η (345) = 0.11, p = .796	η (345) = 0.08, p = .886	χ²(1) = 3.40, p = .065	χ²(3) = 1.721, p = .632	χ²(1) = 0.99, p = .318
Shortness of breath - 30 + days after violent dispersion of the demonstration	η (345) = 0.10, p = .401	η (345) = 0.01, p = .834	χ²(1) = 3.1, p = .078	χ²(3) = 0.770, p = .857	χ²(1) = 0.19, p = .665
Rhinorrhea - 30 + days after violent dispersion of the demonstration	η (345) = 0.12, p = .705	η (345) = 0.13, p = .811	χ²(1) = 0.01, p = .944	χ²(3) = 4.15, p = .246	χ²(1) = 1.30, p = .254
Epistaxis - 30 + days after violent dispersion of the demonstration	η (345) = 0.06, p = .265	η (345) = 0.01, p = .073	χ²(1) = 3.23, p = .072	χ²(3) = 3.23, p = .357	χ²(1) = 0.03, p = .866
Nausea - 30 + days after violent dispersion of the demonstration	η (345) = 0.15, p = .800	η (345) = 0.13, p = .013	χ²(1) = 0.00, p = .994	χ²(3) = 1.51, p = .680	χ²(1) = 0.04, p = .849
Vomiting - 30 + days after violent dispersion of the demonstration	η (345) = 0.00, p = .994	η (345) = 0.02, p = .660	χ²(1) = 0.11, p = .746	χ²(3) = 1.94, p = .584	χ²(1) = 0.13, p = .721
Abdominal pain - 30 + days after violent dispersion of the demonstration	η (345) = 0.12, p = .275	η (345) = 0.02, p = .708	χ²(1) = 0.03, p = .866	χ²(3) = 3.05, p = .384	χ²(1) = 0.632, p = .427
Diarrhea - 30 + days after violent dispersion of the demonstration	η (345) = 0.07, p = .339	η (345) = 0.10, p = .070	χ²(1) = 0.97, p = .325	χ²(3) = 3.14, p = .370	χ²(1) = 1.73, p = .188
Headache - 30 + days after violent dispersion of the demonstration	η (345) = 0.09, p = .412	η (345) = 0.07, p = .193	χ²(1) = 0.00, p = .996	χ²(3) = 4.64, p = .200	χ²(1) = 1.38, p = .239
High blood pressure - 30 + days after violent dispersion of the demonstration	η (345) = 0.01, p = .798	η (345) = 0.02, p = .730	χ²(1) = 0.12, p = .729	χ²(3) = 2.38, p = .498	χ²(1) = 2.79, p = .095
Heart palpitation - 30 + days after violent dispersion of the demonstration	η (345) = 0.05, p = .386	η (345) = 0.03, p = .639	χ²(1) = 0.06, p = .807	χ²(3) = 5.01, p = .498	χ²(1) = 3.62, p = .057
Fatigue - 30 + days after violent dispersion of the demonstration	η (345) = 0.01, p = .813	η (345) = 0.02, p = .675	χ²(1) = 0.00, p = .983	χ²(3) = 0.24, p = .971	χ²(1) = 1.63, p = .201
Muscle and/or joint pain - 30 + days after violent dispersion of the demonstration	η (345) = 0.00, p = .987	η (345) = 0.01, p = .930	χ²(1) = 0.92, p = .337	χ²(3) = 7.16, p = .067	χ²(1) = 6.13, p = .013
Eye and vission sequale - 30 + days after violent dispersion of the demonstration	η (345) = 0.12, p = .928	η (345) = 0.05, p = .342	χ²(1) = 0.89, p = .346	χ²(3) = 5.71, p = .127	χ²(1) = 0.747, p = .387
Skin Disorders (rash, itching and etc.) - 30 + days after violent dispersion of the demonstration	η (345) = 0.01, p = .732	η (345) = 0.03, p = .624	χ²(1) = 0.62, p = .431	χ²(3) = 2.67, p = .446	χ²(1) = 0.415, p = .519
Psychological sequalae (Anxiety, Panic attacks, PTSD etc.) - 30 + days after violent dispersion of the demonstration	η (345) = 0.09, p = .897	η (345) = 0.02, p = .658	χ²(1) = 0.01, p = .915	χ²(3) = 1.13, p = .770	χ²(1) = 1.67, p = .196

BMI exhibited weak correlations with nausea during violent dispersal, elevated blood pressure in the same context, and nausea persisting 30 or more days after violent dispersal (Table 2).

Smoking status correlated with cough, shortness of breath, and vomiting in the close proximity of violent dispersal (Table 2).

Mask use showed no correlation with any signs or presentations (Table 2)

**Fig. 3:**
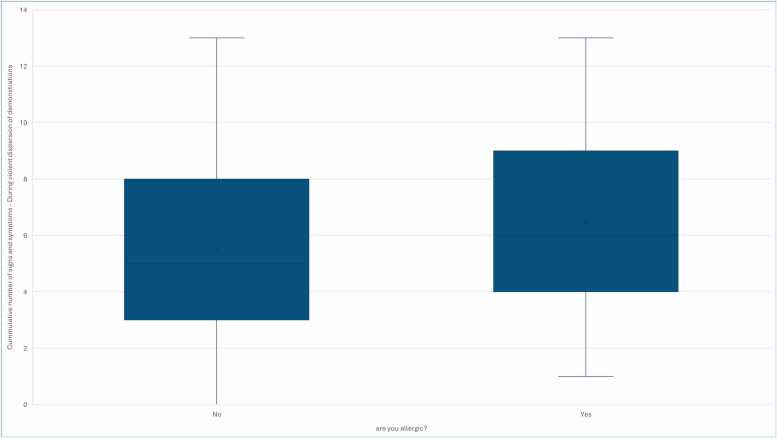
The figure illustrates a relationship between allergy and cummulative number of signs and symptoms in close proximity of chemical exposure.

**Fig. 4:**
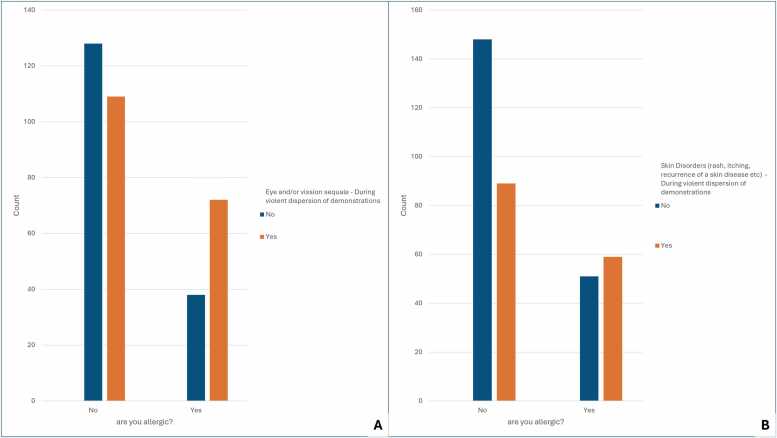
The figure illustrates the relationship between allergy status and/or the presence of eye or visual sequelae (A), and skin-related symptoms and signs (B).

Allergy status correlated with the cumulative number of signs and symptoms (Fig. 3), ocular or visual sequelae, and skin disorders (Fig. 4 ) in close proximity to violent dispersal of demonstrations (Table 2).

### 3.6 Rare cases

Two patients declined formal enrollment but agreed to share their electronic records after having already undergone clinical assessment and diagnosis. One patient had radiographically confirmed left-sided pneumonia that started after exposure to the tear gas and signs and symptoms did not resolve following routine empiric antibiotic therapy. A follow-up chest radiograph performed three weeks after cessation of antibiotics continued to show lower-lobe hyperintensities and increased vascular markings. Spirometry demonstrated mild obstructive impairment, with a 14.2 % improvement in FEV₁ after salbutamol inhalation. Another respondent started to have respiratory symptoms (cough, difficulty breathing, etc) after exposure to the tear gas and diagnosis of hypersensitivity pneumonitis was made: persistent cough and dyspnea led her to a pulmonologist, and pulmonary function tests plus chest CT confirmed the diagnosis. She had started prednisone treatment immediately prior to our data collection. In both cases, symptoms and signs started to show within a week after attending demonstrations where the chemicals were deployed.

## 4 Discussion

Findings indicate a significantly higher prevalence of right bundle branch block (RBBB) and T-wave inversion in the study group, suggesting a potential association with exposure to chemicals deployed during demonstrations. Please, note that there have been widespread claims that pepper spray was mixed into the water used in the water cannons; however, this has not yet been independently verified to date. A plausible mechanism is pulmonary microvascular overload from chemical deposition, leading to impaired oxygenation and increased right ventricular workload; T-wave inversion may reflect myocardial hypoxia. This interpretation is supported by the absence of a significant difference in smoking prevalence between groups, making smoking an unlikely confounder for the observed ECG changes.

The number of days participants reported attending demonstrations with chemical agent deployment did not correlate with the presence or severity of symptoms in our data. This may indicate that residual exposure persisted between active deployments, that a single high-intensity exposure was sufficient to elicit symptoms (given large-scale deployments on some days), also it might indicate that the serious symptoms prompted protestors not to attend further demonstrations or that self-reported attendance data are unreliable. Individuals with a history of allergies showed higher rates of overall symptoms, particularly skin, ocular, and visual disturbances. Mask use and mask type were not associated with symptom incidence, suggesting either improper mask use or that residual environmental contamination before active deployment was sufficient to cause effects.

Chemical irritant exposure can damage skin, mucous membranes, and respiratory tissues through heat, smoke, or irritant compounds. Though often labeled “nonlethal” or “less lethal,” such agents can carry substantial acute and potentially long-term health risks, especially when misused or deployed under real-world conditions. Variability in agent types, concentrations, delivery units, and deployment methods complicates a full understanding of these effects. Typically used for crowd dispersal or individual restraint, irritants may be delivered via targeted sprays or pellets, or in larger-scale deployments (canisters, grenades, water-cannon mixtures) for mass incapacitation [9].

Injury severity depends on the agent, deployment method, environment, and context of use. Aerosolized irritants are indiscriminate, affecting protesters, bystanders, and police alike. Confined or poorly ventilated spaces amplify exposure, while heat and humidity worsen irritation. Vulnerable groups—children, the elderly, and those with chronic disease—face higher risk, and repeated or occupational exposure may cause cumulative toxicity [9], [10].

“Inhalation injury” refers to airway or lung damage caused by irritants, influenced by particle size, duration, and solubility. Lower–molecular-weight gases can reach distal airways, causing upper, tracheobronchial, or parenchymal injury. Manifestations include airway edema, impaired ciliary clearance, cough, atelectasis, and ventilation-perfusion mismatch [11], [12].

The main agents—CS, CN, and CR—provoke ocular, respiratory, and skin irritation within seconds. CS (2-chlorobenzylidene malononitrile) is the most common; its particulate form adheres to moist mucosa and skin, with symptoms such as burning, cough, headache, dyspnea, and chest tightness. High-dose or enclosed exposures may lead to bronchospasm, pneumonitis, pulmonary edema, or even death [12], [13], [14].

CS acts on TRPA1 and TRPV1 nociceptors, triggering pain, inflammation, and neurogenic effects; inhibitors of TRPA1 reduce tissue swelling and cytokine release in experimental models [6], [14], [15]. Systemic absorption occurs mainly via inhalation, with rapid hepatic metabolism. Immediate effects include dermatitis, conjunctivitis, and respiratory distress; severe cases may involve corneal injury, reactive airway dysfunction, pulmonary edema, or cardiovascular reactions such as tachycardia and transient hypertension [8], [14], [16], [17].

Many studies examine acute effects under controlled exposures in healthy volunteers or animal models, but real-world data remain limited. Available studies are often dated, lack granularity (agent type, exposure time, particle size), and do not account for individual variability or environmental modifiers (weather, terrain). Field exposure concentrations and durations are frequently unknown. No clear evidence links CS exposure to electrocardiographic, capillaroscopic, or routine laboratory abnormalities. Case reports and epidemiological studies reveal lung, skin, and ocular injuries, with chronic morbidity risks in vulnerable individuals. TRPV1/TRPA1-mediated mechanisms tie tear gas injury to pathways involved in acute/chronic pain, cough, asthma, dermatitis, and neurodegeneration; animal models suggest transient receptor potential inhibitors as potential countermeasures [16], [18]. Large-scale deployments have been associated with severe injuries and fatalities due to direct impact of munitions (head/eye trauma, burns) in contexts such as Egypt, Turkey, Bahrain, and Brazil [19]. Epidemiological data (e.g., U.S. Army gas-mask training involving >6000 recruits) show heightened acute respiratory illness risk post-CS exposure, increasing with concentration [20]. Rare case reports describe conditions like desquamative interstitial pneumonia following tear gas exposure [21]. Observational longitudinal studies link mass tear gas use with increased respiratory emergencies and bronchial diseases in vulnerable populations [22]. Historical tolerance experiments document exposure thresholds (e.g., tolerating 5 sec at 442 mg/m³, >90 min at 1.5 mg/m³), with particle size influencing irritation onset and recovery times [10], [18]. While most effects reportedly resolve within minutes after removal from exposure, one possible suggestion would be conclusion stems from limited and potentially misinterpreted research. Given persistent uncertainties about long-term health and environmental impacts, a thorough reevaluation of tear gas safety and comprehensive follow-up studies are warranted.

All of the above considerations must be taken into account when authorities contemplate the use of tear gas or pepper spray against civilians. This is particularly true for tear gas, which is inherently indiscriminate and cannot be directed at specific individuals. Its use in densely populated urban areas—such as Rustaveli Avenue in Tbilisi, where pedestrian and vehicular traffic is heavy, numerous offices and institutions are located, and a school sits adjacent to the Parliament building—poses serious risks. In multiple documented instance, this area was saturated with tear gas canisters, some of which landed near the school (supplemental file 1–3). The following morning, children were expected to return to classes despite the lingering chemical residue. Given the known heightened vulnerability of children to tear gas, such use raises major public health and ethical concerns.

In the European Union, there is no unified law governing the use of tear gas. However, EU countries adhere to the UN Basic Principles on the Use of Force, which stipulate that tear gas may only be used when strictly necessary, proportionate, and as a measure of last resort—specifically in response to widespread violence when targeted measures have proven ineffective. Oversight is provided by both national internal police accountability mechanisms and supranational bodies such as the European Court of Human Rights (ECtHR), which acts as a critical human rights watchdog; The United Kingdom follows a similar approach. Law enforcement officers are permitted to use CS or PAVA spray to subdue individuals who pose a risk to themselves, officers, or the public, particularly during serious public disorder. However, pepper sprays are generally favored due to their more targeted application. Oversight is provided by the Independent Office for Police Conduct (IOPC); In the United States, regulation of tear gas varies significantly by state. Some states have adopted stricter legal frameworks limiting its use to specific circumstances such as a declared riot, a barricaded suspect, or a hostage situation. Oversight is conducted through internal affairs departments, civilian review boards, and the judiciary [23], [24], [25], [26], [27], [28], [29], [30], [31]. In contrast, countries like Georgia have, at times, deployed tear gas in the absence of violent unrest, imminent threats, or riot conditions. In such instances, the use of tear gas appears to serve more as a demonstration of political power than as a response to a legitimate security threat, raising serious legal and human rights concerns—particularly in the absence of effective checks and balances, which precludes meaningful oversight or impartial investigation.

It must also be emphasized that the deployment of tear gas is not solely a legal or political matter—it is also a significant public health issue. Repeated or excessive exposure increases the risk of acute and chronic respiratory problems, ocular injuries, and psychological distress, particularly among vulnerable populations such as children, the elderly, and those with preexisting health conditions.

Although observational studies are important and valuable for identifying associations and generating hypotheses, they also have notable limitations. The absence of random assignment makes them vulnerable to confounding variables, limiting the ability to infer causality. When based on surveys, they often rely on self-reported data, which may introduce recall bias or misclassification. A purposive sampling approach was used due to the politically sensitive context of data collection; however, this method introduces selection bias and may be subject to unmeasured confounding variables, meaning the findings should be interpreted with caution. Additionally, as noted in the methodology section, the sample size limited our ability to detect smaller statistical differences or correlations. Therefore, results from the study should be interpreted with these limitations in mind, and conclusions drawn with appropriate caution.

## 5 Conclusion

We identified a significantly higher prevalence of electrocardiographic abnormalities—particularly right bundle branch block and T-wave inversion—among individuals exposed to chemical irritants during demonstrations, as compared to a control group. Capillaroscopic findings showed increased number of non-specific abnormalities,however, these differences were not statistically significant. Allergy status appeared to correlate with increased symptom burden, whereas mask usage and attendance frequency did not predict symptom frequency, suggesting either improper use or pervasive environmental contamination. Although laboratory parameters remained largely within reference ranges, the clinical presentations and rare severe cases underscore the potential for acute and possibly lasting health consequences. Given that tear gas and similar agents are often classified as “non-lethal,” their indiscriminate deployment in urban, densely populated areas raises significant public health, ethical, and legal concerns—particularly for vulnerable populations such as children and individuals with pre-existing conditions. In the end, observational nature of this study limits causal inference, and reliance on self-reported exposure introduces potential recall bias. Nevertheless, these findings warrant urgent reconsideration of riot-control policies and emphasize the need for independent oversight, stricter deployment protocols, and further longitudinal research to evaluate long-term health effects of chemical crowd-control agents.

## CRediT authorship contribution statement

**Davit G. Chakhunashvili:** Writing – review & editing, Writing – original draft, Project administration, Methodology, Investigation, Formal analysis, Data curation, Conceptualization. **George Chakhunashvili:** Writing – review & editing, Writing – original draft, Formal analysis, Data curation, Conceptualization. **Nino Jobava:** Writing – review & editing, Writing – original draft, Data curation, Conceptualization. **Gela Gunashvili:** Writing – review & editing, Writing – original draft, Resources, Investigation, Formal analysis, Conceptualization. **Konstantine Chakhunashvili:** Writing – review & editing, Writing – original draft, Supervision, Resources, Project administration, Methodology, Investigation, Formal analysis, Data curation, Conceptualization.

## Authors' contributions

All authors were involved in at least one of the following: [conception, design of work or acquisition, analysis, interpretation of data] and [drafting the manuscript and/or revising/reviewing the manuscript for important intellectual content]. All authors provided final approval of the version to be published. All authors agree to be accountable for all aspects of the work in ensuring that questions related to the accuracy or integrity of any part of the work are appropriately investigated and resolved.

## Consent for publication

None.

## Ethics approval and consent to participate

The study was approved by University of Georgia ethics committee (#UGREC-01–25) and was conducted in accordance with the Declaration of Helsinki. The participants were informed about the study background, aims. Informed consent was collected from all the patients. All the data was collected anonymously, and confidentiality of the responses was guaranteed by the study team

## Clinical Trial Number

Clinical Trial Number: not applicable

## Authorship

All named authors meet the International Committee of Medical Journal Editors (ICMJE) criteria for authorship for this article, take responsibility for the integrity of the work as a whole, and have given their approval for this version to be published.

## Funding

No financial assistance was received to support the study.

## Declaration of Competing Interest

The authors declare that they have no known competing financial interests or personal relationships that could have appeared to influence the work reported in this paper.

## Data Availability

Data will be made available on request.

## Appendix A Supplementary material

Video clips showing active deployment of tear gas and water cannons on protestors, including footage of tear gas canisters being fired directly into crowds. The video footage documents the use of water cannons and tear gas for crowd dispersal, including instances where tear gas canisters were fired directly into the crowd

Video clips showing active deployment of tear gas and water cannons on protestors, including footage of tear gas canisters being fired directly into crowds. The video footage documents the use of water cannons and tear gas for crowd dispersal, including instances where tear gas canisters were fired directly into the crowd

Video clips showing active deployment of tear gas and water cannons on protestors, including footage of tear gas canisters being fired directly into crowds. The video footage documents the use of water cannons and tear gas for crowd dispersal, including instances where tear gas canisters were fired directly into the crowd

## Footnotes

Appendix A Supplementary data associated with this article can be found in the online version at doi:10.1016/j.toxrep.2025.102166.
